# Scientific hypothesis generation by large language models: laboratory validation in breast cancer treatment

**DOI:** 10.1098/rsif.2024.0674

**Published:** 2025-06-04

**Authors:** Abbi Abdel-Rehim, Hector Zenil, Oghenejokpeme Orhobor, Marie Fisher, Ross J. Collins, Elizabeth Bourne, Gareth W. Fearnley, Emma Tate, Holly X. Smith, Larisa N. Soldatova, Ross King

**Affiliations:** ^1^ Department of Chemical Engineering and Biotechnology, University of Cambridge, Cambridge, UK; ^2^ Algorithmic Dynamics Lab, Research Departments of Biomedical Computing and Digital Twins, School of Biomedical Engineering and Imaging Sciences, King's Institute for AI, King's College London, London, England, UK; ^3^ Oxford Immune Algorithmics, Oxford University Innovation and London Institute for Healthcare Engineering, London, England, UK; ^4^ Cancer Interest Group, The Francis Crick Institute, London, England, UK; ^5^ Defence and National Security, The Alan Turing Institute, British Library, London, England, UK; ^6^ Arctoris Ltd, Oxford, UK; ^7^ Computer Science, Goldsmiths University of London, London, UK; ^8^ Department of Computer Science and Engineering, Chalmers University, Gothenburg, Sweden

**Keywords:** machine learning, personalized medicine, artificial intelligence for science, cancer research, drug discovery

## Abstract

Large language models (LLMs) have transformed artificial intelligence (AI) and achieved breakthrough performance on a wide range of tasks. In science, the most interesting application of LLMs is for hypothesis formation. A feature of LLMs, which results from their probabilistic structure, is that the output text is not necessarily a valid inference from the training text. These are termed ‘hallucinations’, and are harmful in many applications. In science, some hallucinations may be useful: novel hypotheses whose validity may be tested by laboratory experiments. Here, we experimentally test the application of LLMs as a source of scientific hypotheses using the domain of breast cancer treatment. We applied the LLM GPT4 to hypothesize novel synergistic pairs of US Food and Drug Administration (FDA)-approved non-cancer drugs that target the MCF7 breast cancer cell line relative to the non-tumorigenic breast cell line MCF10A. In the first round of laboratory experiments, GPT4 succeeded in discovering three drug combinations (out of 12 tested) with synergy scores above the positive controls. GPT4 then generated new combinations based on its initial results, this generated three more combinations with positive synergy scores (out of four tested). We conclude that LLMs are a valuable source of scientific hypotheses.

## 1. Introduction

The world has been stunned by the success of large language models (LLMs) [1]. They have achieved breakthrough performance on a wide range of conversation-based tasks that previously required human intelligence [2]. The overall architecture of LLMs is remarkably simple: they map input token strings to output token strings using deep neural networks [3]. Their power comes from being trained on very large general corpuses (substantial percentages of the whole text-based Internet), and the use of very large numbers of both tokens (greater than 10^4^) and parameters (greater than 10^12^). The success of LLMs is surprising given that they do not use any explicit model of the world, nor explicit internal symbols, nor do they have any physical grounding in the world. All of these were assumed by most artificial intelligence (AI) scientists to be essential for such intelligent behaviour.

LLMs can be applied to many aspects of science: to summarize texts [4,5], to analyse data [6], to write papers and code [7], to formalize knowledge [8], to answer questions [9], etc. However, the most exciting application for LLMs in science is for generating novel hypotheses. Despite the clear potential of LLMs for hypothesis generation, their utility for hypothesis generation has been little investigated.

Due to their probabilistic nature, LLMs have the potential to go beyond existing text-based scientific hypothesis generation tools [10,11]. The architecture of LLMs entails that the output string is the most likely one given the input string and the training data. The validity of such outputs may be uncertain or even factually wrong—the phenomena of ‘hallucinations’. Hallucinations are a serious problem in many applications [12–14].

In science, it is not acceptable to hallucinate facts and inferences. For example, LLMs can generate misleading insights from datasets by fabricating correlations or causal relationships that do not exist [15], or it can supply false references [16]. However, in scientific hypothesis generation, hallucinations can be exploited, and the validity of probable hypotheses can be objectively tested by laboratory experiments [17]. This is consistent with a regular scientific discovery process that starts with considering a hypothesis which may be true or not [18]. Hypotheses can be formulated by human scientists (e.g. eureka phenomenon) or generated by computational methods. In this article, we consider how LLMs can be utilized for scientific hypothesis generation. Our aim is to identify the advantages, disadvantages and challenges of using LLMs in drug discovery, and to explore their potential to aid scientists in uncovering new cancer treatments.

## 2. Methods

### 2.1. Application domain

**Figure 1.**
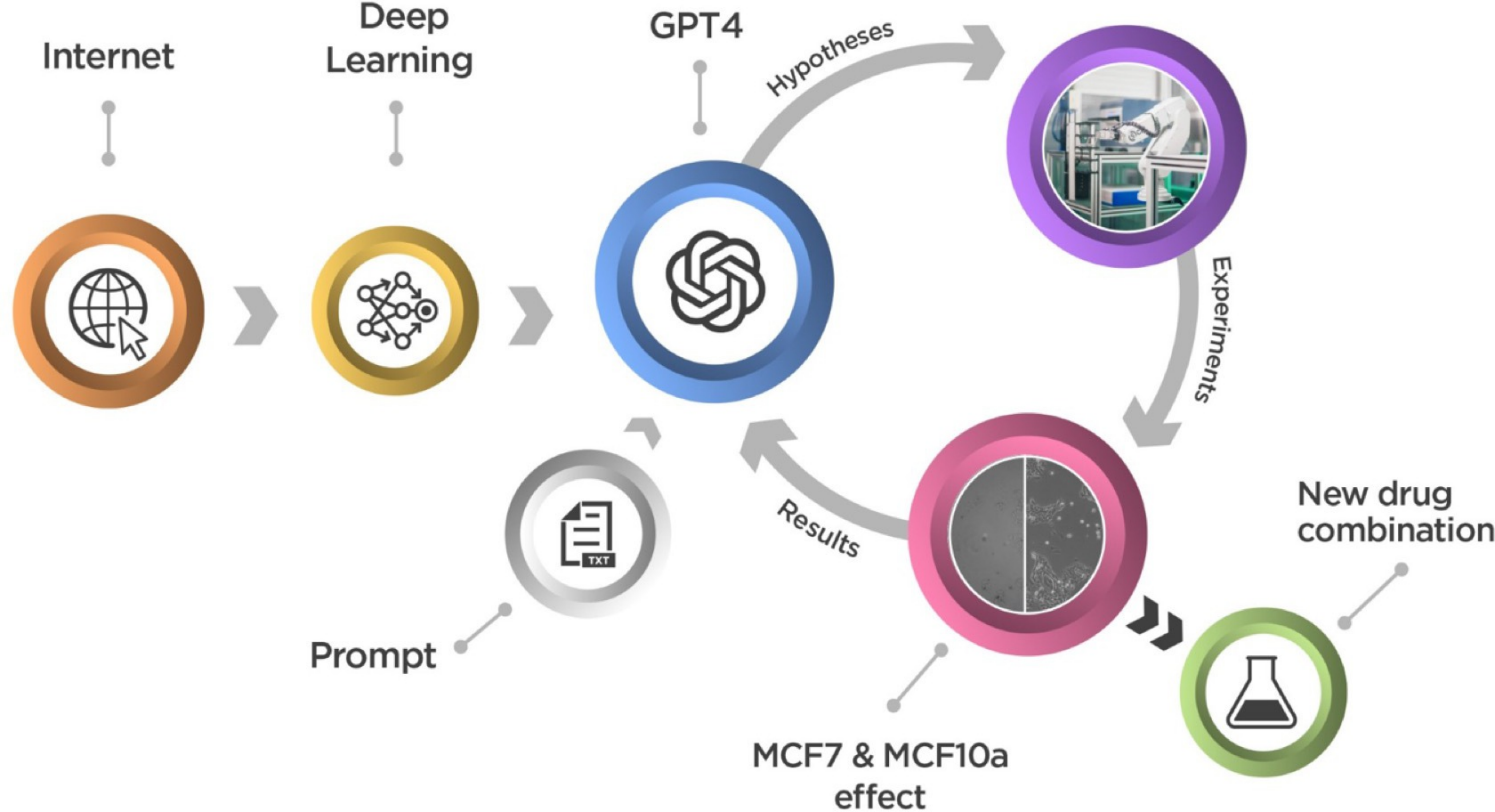
The overall structure of our experiments. GPT4 was previously trained on data on a large fraction of the text on the Internet. We engineered prompts to request from GPT4 synergistic pairs of drugs that are toxic to the breast cancer cell line MCF7, but not to the non-cancer breast cell line MCF10a. These are the hypotheses that we experimentally tested using laboratory automation.

We employed the general purpose LLM GPT4 to generate scientific hypotheses and then run laboratory experiments to test the validity of generated hypotheses (figure 1). We chose breast cancer as our test domain due to its critical importance in medical research, the vast body of existing literature, and our access to specialized equipment for studying tissue cultures as proxies for real patient tumours.

Breast cancer is the most diagnosed cancer and the leading cause of cancer-related deaths among females, accounting for 23% of all cancer cases. Breast cancer is not a single disease but a collection of molecularly distinct subtypes. Treatment options for breast cancer include various drug therapies. However, tumour cells often develop resistance, making treatment less effective over time. A promising strategy to counteract this resistance is the use of drug combinations or ‘cocktails’. While some drugs can enhance each other’s efficacy, others may interfere with one another, complicating treatment strategies. Unfortunately, there is limited data on these interactions and their overall effectiveness. To address this challenge, we leveraged the power of LLMs to analyse drug combinations. By utilizing the knowledge embedded in GPT4, we aimed to explore the regions of the hypothesis space that human researchers might overlook or find difficult to investigate, potentially uncovering novel therapeutic strategies.

### 2.2. Experimental set-up

We prompted ChatGPT4 to propose hypotheses relating to novel drug combinations for targeted breast cancer treatment, specifically towards the MCF7 breast cancer cell line. It is a commonly used breast cancer cell line and has been promoted for more than 40 years as a suitable model cell line for breast cancer investigations.

In our experiments, breast cancer cells were exemplified by MCF7 (an epithelial breast cancer cell line); non-tumorigenic breast cells were exemplified by the epithelial cell line MCF10A. We provided GPT4 with a prompt that had several aims:

1. Identify novel drug combinations that would have a significant impact on MCF7 cell lines.
2. Avoid harming MCF10A, the control cell line.
3. Design combinations that were possibly synergistic.

We also had additional requirements related to the drugs themselves:

1. At least one of the drugs in every pair should not be an antineoplastic drug.
2. The drugs should be affordable, accessible and preferably US Food and Drug Administration (FDA) approved.

Antineoplastic drugs are medications used to treat cancer by preventing the growth and spread of tumours. Unfortunately, they are associated with potential risks to pregnant women and can cause adverse effects on reproductive health.

Prompts and the list of complete hypotheses are found in electronic supplementary material, figures S1–S3 and table S1. Interestingly, all the drug combinations hypothesized were exclusively non-cancer drugs (suggesting a possible limitation in GPT4’s understanding of its instructions). We could not find in the cancer literature any of the generated combinations (electronic supplementary material, appendix B). We found that several individual drugs had been tested against MCF7, but not combinations. Therefore, the generated drug combinations were novel.

In addition to hypothesizing drug combinations, we prompted GPT4 to provide two positive controls that are commonly used against breast cancer in clinics and likely have an impact on MCF7, as well as two negative control combinations that would be unlikely to cause harm to MCF7 (electronic supplementary material, figures S2 and S3). It may have been wiser to select the controls ourselves, but we judged that GPT4 did a fair job in its selections (table 1b).

**Table 1.** GPT4 generated drug combination hypotheses.

a)	drug1	drug2
1	disulfiram (alcoholism)	simvastatin (hypercholesterolemia)
	‘disruption of lipid rafts by simvastatin may enhance disulfiram-induced oxidative stress, leading to apoptosis in MCF7 cells’.	
2	celecoxib (pain/inflammation)	quinacrine (malaria/anti-inflammatory)
	‘reduced inflammatory signalling by celecoxib may enhance quinacrine-induced impairment of autophagy, leading to apoptosis in MCF7 cells’.	
3	acarbose (diabetes)	itraconazole (fungal infections)
	‘acarbose-induced glucose deprivation may enhance the effect of itraconazole on disrupting cell membrane integrity, leading to apoptosis in MCF7 cells’.	
4	dipyridamole (blood thinner)	mebendazole (parasitic infections)
	‘dipyridamole-induced increase in cAMP levels may enhance the effect of mebendazole on cell cycle arrest, leading to apoptosis in MCF7 cells’.	
5	atorvastatin (hypercholesterolemia)	metronidazole (bacterial infections)
	‘atorvastatin-induced disruption of lipid rafts may enhance the effect of metronidazole-induced DNA damage, leading to apoptosis in MCF7 cells’.	
6	allopurinol (gout)	chloroquine (malaria)
	‘allopurinol-induced reduction of uric acid may enhance the effect of chloroquine-induced impairment of autophagy, leading to apoptosis in MCF7’.	
7	cimetidine (gastric acid reducer)	disulfiram (alcoholism)
	‘cimetidine-induced reduction of gastric acid may enhance the effect of disulfiram-induced oxidative stress and DNA damage in MCF7 cells’.	
8	memantine (Alzheimer's)	niclosamide (parasitic infections)
	‘memantine-induced reduction of glutamate excitotoxicity may enhance the effect of niclosamide-induced energy depletion, leading to cell death in MCF7 cells’.	
9	furosemide (diuretic)	mebendazole (parasitic infections)
	‘furosemide-induced reduction of sodium reabsorption may enhance the effect of mebendazole on cell cycle arrest, leading to apoptosis in MCF7 cells’.	
10	sildenafil (erectile dysfunction)	disulfiram (alcoholism)
	‘sildenafil-induced increase in cGMP levels may enhance the effect of disulfiram-induced oxidative stress and DNA damage in MCF7 cells.’	
11	itraconazole (fungal infections)	atenolol (hypertension)
	‘atenolol-induced reduction of stress signaling may enhance the effect of itraconazole on disrupting cell membrane integrity, leading to apoptosis in MCF7 cells’.	
12	hydroxychloroquine (malaria, rheumatoid arthritis)	disulfiram (alcoholism)
	‘hydroxychloroquine-induced impairment of autophagy may enhance the effect of disulfiram-induced oxidative stress and DNA damage in MCF7 cells’.	

b)	drug1	drug2
1	doxorubicin	cyclophosphamide
	positive control combination FDA approved to treat breast cancer doxorubicin is an anthracycline that intercalates into DNA and inhibits topoisomerase II, causing DNA damage. Cyclophosphamide is an alkylating agent that causes DNA damage. ‘the combination targets DNA integrity through multiple mechanisms, which may be effective in MCF7 cells with high proliferative capacity’.	
2	fulvestrant	palbociclib
	positive control individually FDA approved to treat breast cancer fulvestrant is a selective oestrogen receptor degrader (SERD) that blocks and degrades oestrogen receptors. Palbociclib is a CDK4/6 inhibitor that blocks cell cycle progression. ‘the combination targets both oestrogen signalling and cell cycle progression, which may be effective in oestrogen receptor-positive MCF7 cells’.	
3	allopurinol (xanthine oxidase inhibitor)	omeprazole (proton pump inhibitor)
	negative control ‘allopurinol is used to treat gout and hyperuricemia, and omeprazole is used to reduce stomach acid. Neither drug targets pathways relevant to MCF7 breast cancer cell growth or survival, and they are not expected to have an effect on MCF7 cells’.	
4	diphenhydramine (antihistamine)	omeprazole (proton pump inhibitor)
	negative control ‘diphenhydramine and cetirizine are antihistamines used to treat allergy symptoms. Neither drug targets pathways relevant to MCF7 breast cancer cell growth or survival, and they are not expected to have an effect on MCF7 cells’.	

## 3. Results

### 3.1. Hypotheses generation: first iteration

Using the method described below, we screened the 12 pairs of compounds proposed by GPT4 (table 1a). We investigated two properties of the pairs:

1. The specificity of the combination for MCF7 versus MCF10A.
2. The additivity/synergy of the combination.

Additivity occurs when the combination of the effects of two drugs is not less than either of the two drugs acting independently. Synergy describes the situation when the effect of the combination is greater than that of the most effective drug (highest single agent; HSA).

To determine drug additivities/synergies, we employed SynergyFinder 3.0 to calculate HSA synergy scores for all combinations (table 2). There were six additive combinations with positive synergy scores for MCF7: itraconazole + atenolol, simvastatin + disulfiram, dipyridamole + mebendazole, furosemide + mebendazole, disulfiram + hydroxychloroquine and the positive control doxorubicin + cyclophosphamide. The initial three hypothesized combinations resulted in HSA scores surpassing those of the positive controls. Synergistic areas were found within the drug response matrices belonging to 10 out of 12 of the hypothesized drug combinations (electronic supplementary material, table S2). We found that 8 out of the 12 hypothesized combinations resulted in a higher HSA score in varying degrees for MCF7 compared with MCF10A (table 2; cf. table 2 and electronic supplementary material, table S3). In electronic supplementary material, table S12, we summarize the literature on the hypotheses proposed by GPT4 and the anti-cancer properties of the drugs selected. We found underlying support in literature for three out of the latter screened six combinations with positive synergy scores, while the remaining three remain unclear.

**Table 2.** HSA synergy scores for tested combinations.

drug 1	drug 2	HSA score (MCF7)	specificity (MCF7)
itraconazole	atenolol	4.83	7.03
simvastatin	disulfiram	3.29	1.85
dipyridamole	mebendazole	2.49	3.69
doxorubicin*	cyclophoshamide*	1.02	3.27
furosemide	mebendazole	0.72	6.14
disulfiram	hydroxychloroquine	0.60	3.51
acarbose	itraconazole	−1.36	−1.33
disulfiram	sildenafil	−1.63	0.85
allopurinol	chloroquine	−1.87	2.24
celecoxib	quinacrine	−2.21	−3.27
fulvestrant*	palbociclib*	−2.59	−0.49
memantine	niclosamide	−2.61	−2.23
disulfiram	cimetidine	−3.06	−8.17
allopurinol**	omeprazole**	−3.85	−6.2
atrovastatin	metronidazole	−4.84	−6.3
ddiphenhydramine**	cetirizine**	−9.25	−6.28

*Positive controls, **negative controls

To better understand the utility of the paired compounds, we tested the individual drugs (table 3). From the drugs in the positive controls pairs, only doxorubicin was found to result in an IC_50_ value below the maximum dose of 25 µM in both cell lines. For MCF7, there were five additional drugs that resulted in IC_50_ values below the same threshold, with disulfiram and niclosamide showing comparatively high toxicity (table 3). Several more drugs were toxic to the cell lines, but failed to reduce the viability to such an extent, where IC_50_ value could be derived (electronic supplementary material, tables S4 and S5). In total, 12 out of the 18 non-control drugs showed toxicity towards MCF7: celecoxib, cimetidine, chloroquine, dipyridamole, disulfiram, hydroxychloroquine, itraconalzole, mebendazole, niclosamide, quinacrine, sildenafil and simvastatin. Out of these drugs, dipyridamole, disulfiram, mebendazole and quinacrine showed high specificity towards MCF7 (cf. electronic supplementary material, tables S4 and S5). While many of these drugs had been studied in cancer cell lines, they are not cancer drugs. Fulvestrant, a positive control cancer drug also showed preference for MCF7.

**Table 3.** Single drug treatments.

drug	MCF7 IC_50_ (µM)	MCF7 *p*-value	MCF10A IC_50_ (µM)	MCF10A *p*-value
allopurinol**	>25	—	>25	—
atenolol	>25	**0.003**	>25	0.118
celecoxib	5.325	**0.046**	22.573	0.185
disulfiram	0.204	**0.008**	>25	0.095
fulvestrant*	>25	**0.020**	>25	0.430
itraconazole	>25	**0.021**	>25	0.077
sildenafil	>25	**0.011**	>25	0.212
cimetidine	>25	**0.012**	>25	**0.023**
mebendazole	>25	**0.025**	15	**0.018**
metronidazole	>25	**0.039**	>25	**0.031**
atorvastatin	>25	0.131	3.795	**0.009**
chloroquine	>25	0.202	>25	**0.030**
doxorubicin*	0.303	0.054	0.435	**0.034**
memantine	>25	0.834	>25	**0.022**
niclosamide	0.699	0.066	0.061	**0.021**
acarbose	>25	0.251	>25	**0.019**
cetirizine**	>25	0.210	>25	0.257
cyclophosphamide*	>25	0.276	>25	0.499
diphenhydramine**	>25	0.684	>25	0.500
dipyridamole	>25	0.056	>25	0.093
furosemide	>25	0.246	>25	0.188
hydroxychloroquine	>25	0.118	>25	0.944
omeprazole**	>25	0.082	>25	0.245
palbociclib*	>25	0.414	>25	0.650
quinacrine	3.848	0.082	10.183	0.116
simvastatin	5.634	0.106	7.17	0.120

*Positive controls, **negative controls. Bold values indicate *p* < 0.05, which is considered significant.

Eleven out of 18 compounds reduced the viability of the control cell line MCF10A. When excluding the highest concentration of 25 µM, these numbers change to 6/18 and 8/18 compounds for MCF7 and MCF10A, respectively. The 10 drugs that showed highest toxicity towards MCF7 were re-screened to achieve sufficient replicates (*n* = 3) in order to validate their toxicity.

In an additional experiment, 12 drugs were retested from the first round (*n* ≥ 3 replicates). An ANOVA two-way test with three replicates was used to calculate the significance of changes to viability compared with the internal control drug allopurinol for both cell lines (electronic supplementary material, figures S6 and S7). Two of the retested drugs were initially used as positive controls for MCF7 (doxorubicin and fulvestrant). Out of 12 retested drugs, dipyridamole, disulfiram, niclosamide and quinacrine significantly reduced the viability of MCF7 when considering concentrations up to 3.84 µM. The two positive control drugs, doxorubicin and fulvestrant, also targeted MCF7. When considering all concentrations (including 25 µM which is quite high), all but hydroxychloroquine results in a significant impact on MCF7. Despite this, the toxicity of hydroxychloroquine at 25 µM is persistent and substantial. IC_50_ values could only be calculated for 5/11 compounds (electronic supplementary material, table S8), disulfiram and doxorubicin showing submicromolar IC_50_ values of 0.059 and 0.3 µM, respectively. This was followed by niclosamide at 1.22 µM, quinacrine at 4.71 µM and chloroquine at 10.62 µM. However, the remaining compounds were still toxic to MCF7 (electronic supplementary material, table S9). Calculated IC_50_, and viability values for MCF10A can be found in electronic supplementary material, tables S8 and S10.

### 3.2. Hypotheses generation: second iteration

After the first round of experiments, we investigated whether GPT4 could improve its hypotheses through the use of the results from its initial hypotheses. We provided GPT4 with a summary of the results from the primary screen (electronic supplementary material, figure S4) and prompted GPT4 to consider combinations containing drugs from the positive controls as well. GPT4 hypothesized four combinations based on this information: disulfiram + fulvestrant, disulfiram + mebendazole, mebendazole + quinacrine and disulfiram + quinacrine (table 4). In addition, we retested three combinations that resulted in positive synergy scores from the primary screening achieving more robust results, these combinations were disulfiram + simvastatin, disulfiram + hydroxychloroquine and dipyridamole + mebendazole. Out of the seven combinations screened in the second iteration, six combinations showed varying degrees of synergy within the response matrices (table 5). Of the newly hypothesized pairs, we found three pairs with positive synergy scores: mebendazole + quinacrine, disulfiram + fulvestrant and disulfiram + quinacrine. The remaining three retested combinations also showed consistent positive scores. The three combinations with the highest HSA scores also showed specificity (greater than 1 HSA score) towards MCF7. It is worth mentioning that the most synergistic 3 × 3 dose–response window resulted in one of the combinations (disulfiram + simvastatin) having a synergy score greater than 10. HSA scores for MCF10A can be found in electronic supplementary material, table S11. Plots showing HSA synergy graphs for MCF7 and MCF10A derived from Synergyfinder 3.0 can be found in electronic supplementary material, appendix D1 and D2.

**Table 4.** GPT4 drug combination recommendations based on results from the first screen.

	drug1	drug2	
1	disulfiram (alcoholism)	fulvestrant (breast cancer)	+
	‘disulfiram exhibited the highest toxicity to MCF7 cells, and both disulfiram and fulvestrant preferentially targeted MCF7 over MCF10A. MCF7 is an oestrogen-receptor positive cell line, and fulvestrant is an oestrogen-receptor antagonist, which degrades the receptor. This combination could target the oestrogen-dependent growth of MCF7 cells. Disulfiram's aldehyde dehydrogenase inhibitory action can further increase its toxicity to MCF7 cells’.		
2	mebendazole (parasitic infections)	quinacrine (malaria/anti-inflammatory)	+
	‘both mebendazole and quinacrine preferentially targeted MCF7 cells. Mebendazole has anti-cancer properties, potentially by disrupting microtubule function. Quinacrine, though primarily an antimalarial, can exert anti-cancer effects, possibly due to its ability to intercalate with DNA and/or inhibit topoisomerase. This combination might have a multi-pronged effect on MCF7 cells, disrupting cellular division and DNA processes’.		
3	disulfiram (alcoholism)	mebendazole (parasitic infections)	—
	‘as disulfiram demonstrated the highest toxicity to MCF7 cells and mebendazole also preferentially targeted MCF7, their combination might exert a compounded effect. The combined microtubule disruption by mebendazole and the aldehyde dehydrogenase inhibition by disulfiram could cripple the MCF7 cells at multiple fronts’.		
4	disulfiram (alcoholism)	quinacrine (malaria/anti-inflammatory)	+
	‘disulfiram exhibited the highest toxicity toward MCF7 cells and has been shown to induce oxidative stress and inhibit proteasomal and NF-κB pathways, which are vital for cancer cell survival. Quinacrine, an antimalarial drug, also possesses anti-cancer properties, including the ability to inhibit NF-κB and autophagy, a survival mechanism often upregulated in cancer cells. The combination is expected to potentiate oxidative stress and inhibit survival pathways more effectively in MCF7 cells. Given that both drugs preferentially targeted MCF7 over MCF10A, their combination might enhance selectivity, exploiting their shared mechanisms for a synergistic effect’.		

**Table 5.** HSA synergy scores for each combination in the second screen.

drug 1	drug 2	HSA score	HSA score (max)	specificity
disulfiram	simvastatin	4.75	10.58	2.41
disulfiram	fulvestrant	1.81	4.60	0.03
disulfiram	quinacrine	1.53	4.47	0.6
dipyridamole	mebendazole	1.10	5.26	3.60
disulfiram	hydroxychloroquine	1.08	3.06	−1.86
mebendazole	quinacrine	0.56	5.54	1.09
disulfiram	mebendazole	−2.49	0.13	−1.83

When comparing the most synergistic dose–response windows across both cell lines, there were three combinations that showed substantially higher synergy against MCF7: quinacrine + mebendazole (ΔHSA = 2.73), mebendazole + dipyridamole (ΔHSA = 3.99) and simvastatin + disulfiram (4.01) (cf. table 5 and electronic supplementary material, table S11). In addition, the two latter combinations also showed areas of selective toxicity towards the MCF7 cell line (electronic supplementary material, appendix D1 and D2).

### 3.3. Hypotheses generation: final iteration

A final query was made to GPT4 requesting future experiments based on the final results (electronic supplementary material, figure S5). Three drug combinations were recommended: disulfiram + itraconazole, mebendazole + cimetidine and quinacrine + celecoxib. Hypotheses for these combinations are reported in electronic supplementary material, table S13. Disulfiram + itraconazole were hypothesized to synergize based on increased oxidative stress and the inhibition of the hedgehog pathway. Mebendazole and cimetidine were also hypothesized to synergize due to their targets being involved in cell cycle progression and growth. The final combination quinacrine + celecoxib had been tested in the initial experiment, suggesting that GPT4 had already ‘forgotten’ its previous recommendations.

### 3.4. Large language models

This study focused on GPT4, but there are multiple other LLMs available. We compared the outputs from GPT4, Gemini and the specialized LLM PubMedGPT. The results revealed both similarities and notable differences in the selected drugs and their subsequent combinations (electronic supplementary material, appendix C). It was evident that LLMs generated non-uniform distributions in their drug suggestions, with certain drugs being consistently selected across models, while pairs exhibit greater variability. Despite this diversity, the suggestions remain consistent in their underlying choices. The specialized LLM PubMedGPT recommended different drug pairs, but with a strong overlap in core drug selections—an aspect that has both advantages and drawbacks. In future studies, it may be useful to analyse pair frequency distributions per LLM.

## 4. Discussion and conclusion

LLMs are already employed by scientists to empower their activities, be it a search of relevant literature, writing code to analyse data, designing experiments, extracting insights from large datasets, or even facilitating interdisciplinary collaboration through natural language translation and knowledge synthesis [10,19]. In this article, we explore how LLMs can be leveraged for hypothesis generation, assessing both their advantages and limitations.

The cost of scientific research traditionally consists of two primary components: the intellectual effort of human scientists and the financial burden of laboratory experiments. With the rapid advancements in AI, the cost of machine-driven scientific intelligence is decreasing. It is inevitable that LLMs will play an increasingly significant role in scientific discovery. We are already witnessing the emergence of AI scientists and AI-assisted researchers, signalling a shift in the way science is conducted.

Our aim is to analyse the advantages, disadvantages and challenges of using LLMs for producing novel scientific hypotheses. Our study of LLM-generated hypotheses relating to novel drug combinations for targeted breast cancer treatment demonstrated several benefits and revealed some limitations of using LLMs.

Benefits:

— By leveraging the vast knowledge encoded in LLMs, scientists can explore regions of the hypothesis space that human researchers may miss or find more difficult to explore due to biases, exhaustion, or other factors.
— Different LLMs, or even the same LLM due to its probabilistic nature, are likely to produce different sets of hypotheses given the same prompts. We tried three different LLMs (electronic supplementary material, appendix C). It is outside of the scope of this study to analyse the variability of such outcomes. Instead, we consider how such variability might be exploited to aid scientists in uncovering new cancer treatments. We argue that the more novel hypotheses are available to test—the better chances for new discoveries.
— LLMs make expertise in drug design (or other application domains) more accessible to non-specialists. It is particularly important for interdisciplinary investigations. For example, in our project, computer scientists are collaborating with bioinformaticians and chemists. Querying LLMs allows non-specialists to produce hypotheses about drug combinations, which then can be screened by experts before investigating further in laboratories.

Limitations:

— It is unclear to what extent GPT4 ‘understood’ its prompt for hypothesis formation. This epistemological uncertainty is shown in the relationship between the explanation of why a pair of drugs would target MCF7 rather than MCF10A (electronic supplementary material, table S1), and the explanation why MCF10A would not be targeted, where the MCF10A hypotheses are simply negations of the MCF7 ones. More convincing explanations for not targeting MCF10A would have provided us with more confidence in GPT4’s understanding, and the utility of its hypotheses.
— LLMs’ outputs are not necessarily consistent with known biological knowledge. Some of ChatGPT4’s explanations for hypotheses were biologically flawed. This is most clearly illustrated by GPT4’s hypothesis that itraconazole would ‘disrupt(ing) cell membrane integrity’. This explanation presumably originated from the fact that itraconazole inhibits ergosterol synthesis, which disrupts cell membrane integrity. The factual error is that ergosterol synthesis is not present in mammalian cells. We asked GPT4 ‘is ergosterol synthesis present in mammalian cells?’. It replied, ‘No, ergosterol synthesis is not present in mammalian cells. Ergosterol is a sterol found in the cell membranes of fungi and some protozoa, playing a role similar to cholesterol in mammalian cells …’.
— One avenue for future investigation is whether using curated literature and/or datasets as opposed to the whole Internet improves the quality of hypotheses.

To conclude, the overall results of this study imply that LLMs are successful at forming novel scientific hypotheses, benefiting both human researchers and the increasingly sophisticated AI systems designed to automate aspects of the scientific process.

## Data Availability

All experimental data are available in the main text or within the electronic supplementary material [20].
